# Fast Homozygosity Mapping and Identification of a Zebrafish ENU-Induced Mutation by Whole-Genome Sequencing

**DOI:** 10.1371/journal.pone.0034671

**Published:** 2012-04-04

**Authors:** Marianne L. Voz, Wouter Coppieters, Isabelle Manfroid, Ariane Baudhuin, Virginie Von Berg, Carole Charlier, Dirk Meyer, Wolfgang Driever, Joseph A. Martial, Bernard Peers

**Affiliations:** 1 Laboratoire de Biologie Moléculaire et de Génie Génétique (LBMGG), Université de Liège, Sart Tilman, Belgium; 2 Giga Geno-transcriptomic Platform, Université de Liège, Sart Tilman, Belgium; 3 Animal Genomics, GIGA-R, Université de Liège, Sart Tilman, Belgium; 4 Institut für Molekularbiologie, Leopold-Franzens-Universität Innsbruck, Innsbruck, Austria; 5 Abteilung Entwicklungsbiologie, Faculty of Biology, Universität Freiburg, Freiburg, Germany; National University of Singapore, Singapore

## Abstract

Forward genetics using zebrafish is a powerful tool for studying vertebrate development through large-scale mutagenesis. Nonetheless, the identification of the molecular lesion is still laborious and involves time-consuming genetic mapping. Here, we show that high-throughput sequencing of the whole zebrafish genome can directly locate the interval carrying the causative mutation and at the same time pinpoint the molecular lesion. The feasibility of this approach was validated by sequencing the *m1045* mutant line that displays a severe hypoplasia of the exocrine pancreas. We generated 13 Gb of sequence, equivalent to an eightfold genomic coverage, from a pool of 50 mutant embryos obtained from a map-cross between the AB mutant carrier and the WIK polymorphic strain. The chromosomal region carrying the causal mutation was localized based on its unique property to display high levels of homozygosity among sequence reads as it derives exclusively from the initial AB mutated allele. We developed an algorithm identifying such a region by calculating a homozygosity score along all chromosomes. This highlighted an 8-Mb window on chromosome 5 with a score close to 1 in the *m1045* mutants. The sequence analysis of all genes within this interval revealed a nonsense mutation in the *snapc4* gene. Knockdown experiments confirmed the assertion that *snapc4* is the gene whose mutation leads to exocrine pancreas hypoplasia. In conclusion, this study constitutes a *proof-of-concept* that whole-genome sequencing is a fast and effective alternative to the classical positional cloning strategies in zebrafish.

## Introduction

The zebrafish (*Danio rerio*) is used extensively to identify genes involved in various aspects of vertebrate development through forward genetic approaches [1], [2]. This process involves random mutagenesis and subsequent isolation of mutants defective in a given process. Although insertional mutagenesis with retroviral vectors has been used in some genetic screens [3], the great majority of zebrafish mutations have been induced by the point mutagen ethylnitrosourea (ENU). The ensuing identification of the molecular lesion in a mutant strain relies on the identification of polymorphic markers genetically linked to the mutation, which requires the establishment of map crosses between the line carrying the mutation (usually the AB, TL or Tü line) and a polymorphic strain (e.g. WIK or SJD). Positional cloning is still laborious and time-consuming and involves three iterative steps [4]. In the first step, bulked mutant embryos and their wild-type siblings are scored by polymerase chain reactions (PCRs) for hundreds of simple sequence length polymorphisms (SSLPs) that cover all chromosomes at approximately equal distances. This allows SSLPs linked to the mutation to be identified and in this way a large chromosomal region spanning the molecular lesion to be located. Second, fine mapping is performed by genotyping thousands of individual mutant embryos for SSLPs present in the identified chromosomal region. Breakpoint mapping allows assignment of the locus of interest to a small interval. Finally, candidate genes within this interval must be identified and sequenced to find the causative mutation.

New high-throughput sequencing technologies show tremendous promise for reducing the time needed to find causative mutations. In *Caenorhabditis elegans* and *Drosophila*, whole-genome sequencing (WGS) of mutants has recently been shown to be an efficient and rapid method to directly identify the causal mutation [5], [6]. Since the zebrafish genome is roughly tenfold larger, mapping by WGS is much more challenging for this organism. However, by identifying the causal lesion in a zebrafish mutant line affected in pancreas formation, we demonstrate here that WGS can also be used in zebrafish to pinpoint the causative mutation.

## Results

### *m1045* mutant isolation and characterization

**Figure 1:**
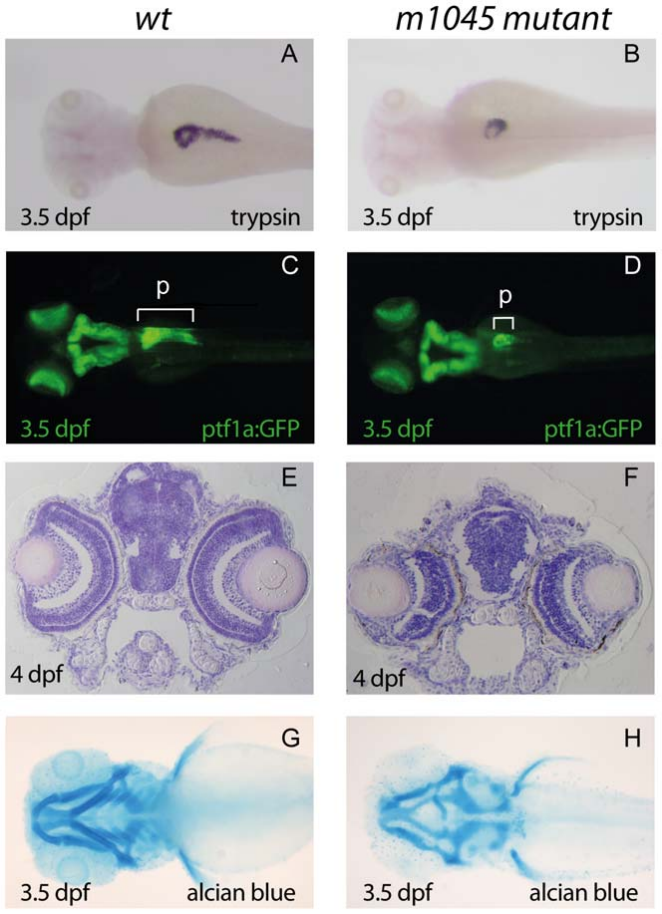
The *m1045* mutant exhibits hypoplasia of exocrine pancreas, eyes and branchial arches. (A,B) : WISH using a *trypsin* probe of unaffected siblings (A) and *m1045* mutant embryos (B) at 3.5 days post fertilization (dpf). (C–D) Dorsal view of fluorescent 3.5 dpf unaffected siblings (C) and *m1045* mutants (D) in the transgenic ptf1∶GFP background. (E,F) Haematoxylin/eosin staining of transverse sections of 4 dpf unaffected siblings (C) and *m1045* mutants (D). Alcian blue staining of the cartilage of 3.5 dpf unaffected siblings (C) and *m1045* mutants (D). A–D, G–H : views are dorsal; anterior part to the left. p: pancreas.

Through an ENU mutagenesis screen to identify mutations affecting pancreas development, we isolated an *m1045* recessive mutant allele characterized by severe pancreatic hypoplasia at 3.5 days post fertilization (dpf) (Figure 1A–B). Before 3 dpf, the homozygous *m1045* mutant larvae were morphologically indistinguishable from the wild-type (wt) siblings (data not shown). From day 3 onwards, the exocrine pancreas of wt larvae undergoes dramatic growth giving rise to the formation of the pancreatic tail, as visualized with the transgenic line *Tg(Ptf1∶GFP)*, which expresses GFP throughout the exocrine tissue as well as in the hindbrain and the retina [7] (Figure 1C). In the *m1045* homozygous mutant, the pancreatic tail did not form (Figure 1D). In contrast, the early stages of pancreas differentiation and morphogenesis appeared unaffected as indicated by the normal expression at 2 dpf of the pancreatic markers mnr2 and ptf1, as well as the early endoderm markers foxA1, foxA2 and foxA3 (data not shown). Moreover, the pancreatic endocrine cells deriving from the dorsal pancreatic bud were not affected, as revealed by the normal expression of insulin, glucagon and somatostatin at 30 hours post fertilization (hpf)(data not shown). Exocrine pancreas was not the only affected tissue as, after 3 dpf, the mutants also displayed markedly smaller eyes and liver as well as an underdeveloped jaw. Haematoxilin/eosin staining of transverse sections of 4 dpf larvae indicated that while all the different retinal layers seemed to be present, they were severely hypoplasic (Figure 1E–F). Alcian blue staining of the cartilage of the jaw revealed that, while the neurocranium seemed well formed in the *m1045* mutant, the viscerocrane was strongly affected (Figure 1G–H). The second branchial arch (i.e. the hyoid) was severely reduced and dysmorphic while the branchial arches 3 to 7 were not detected.

**Figure 2:**
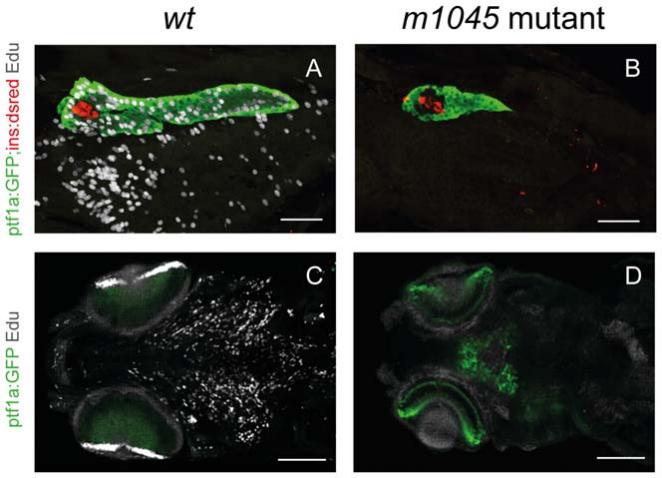
The *m1045* mutant displays a complete loss of cell proliferation in all tissues at 4 dpf. 1-hour Edu incorporation (95–96 hfp) of unaffected siblings (A,C) and *m1045* mutant embryos (B,D) in the transgenic ptf1∶GFP,ins:dsred background. Confocal projections of 4 dpf embryos of the pancreatic region (A,B) (lateral view) and of the head region (ventral view). Scale bars A,B 50 µm; C,D 100 µm. CMZ: ciliary marginal zone.

As these defects affect tissues that undergo a dramatic growth expansion at larval stages, we hypothesized that the observed phenotype could result from cell proliferation defects. Thus, we examined at 3 and 4 dpf the incorporation of the thymidine analogue Edu as a measure of DNA synthesis (Figure 2). While high cell proliferation was detected in the exocrine pancreas of wt larvae (Figure 2A), no cell proliferation could be detected in the *m1045* mutant (Figure 2B). As expected, the insulin cells from both backgrounds were postmitotic. Cell proliferation in the mutant was blocked not only at the level of the exocrine pancreas but also in all tissues of the larvae and notably, no cell proliferation could be detected in the jaw or in the ciliary marginal zone (CMZ) of the eyes, responsible for almost all retinal growth after 60 hours ([8], [9] (Figure 2C–D).

All these data strongly suggest that the hypoplasia of the exocrine pancreas, retina, liver and cartilage results from a blockage in cell proliferation at around 3 dpf in the *m1045* mutant.

### Homozygosity mapping of the *m1045* mutation within a 6.8 Mb interval on chromosome 5

**Figure 3:**
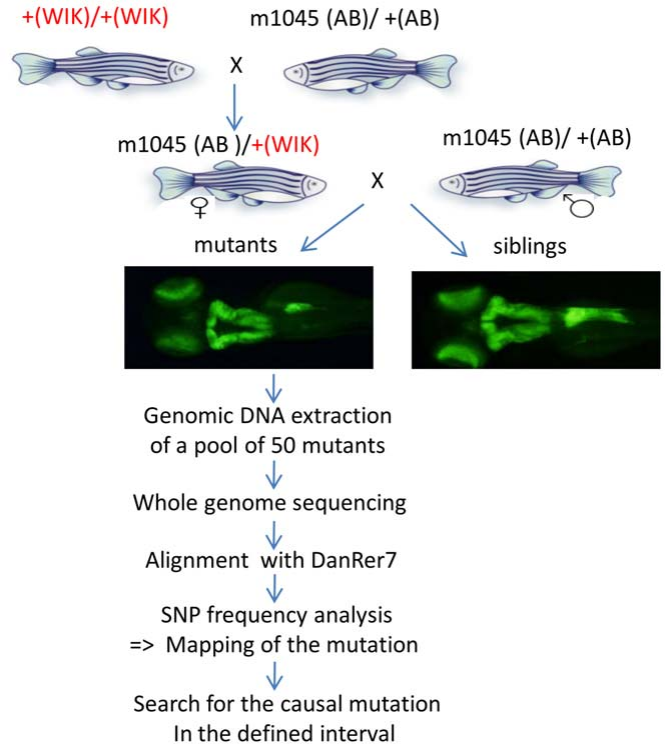
Scheme depicting the strategy used to map and identify the *m1045* mutation.

**Figure 4:**
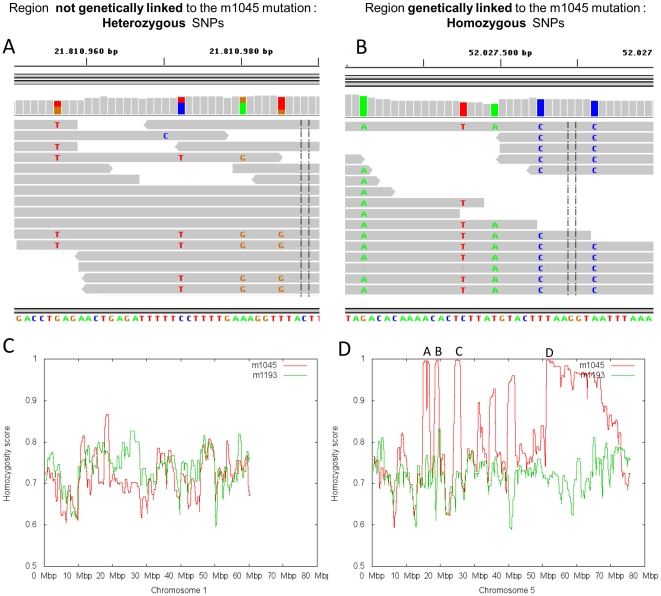
Analysis of the SNP homozygosity scores for the chromosomes 1 and 5. A–B : Visualisation of the sequence reads with the integrative Genomics Viewer [11] of the region 21010950 to 21011990 on chromosome 1, where all SNPs are heterozygous and therefore not genetically linked to the mutation (A) and of the region 52027480 to 52027520 on chromosome 5 where the SNPs are homozygous and linked to the *m1045* mutation (B). SNPs homozygosity score for *m1045* (in red) and for *m1193* (in green) plotted against their respective position on chromosome 1 (C) and on chromosome 5 (D).

In order to map the *m1045* mutation on the zebrafish genome, heterozygous fish for this allele (AB strain) were crossed with the polymorphic strain WIK and subsequently *m1045* (AB)/+ (WIK) females were crossed with *m1045* (AB)/+ (AB) to generate *m1045* homozygous mutant embryos and their siblings (Figure 3). Genomic DNA was isolated from of a pool of 50 mutants and sequenced on 3 lanes using paired-end Illumina sequencing technology (see Material and Methods). In total 12.6 Gb of paired-end 76-mer sequence were generated, resulting in an eightfold coverage of the *m1045* genome after excluding reads that were potential PCR duplicates or failed to map to unique locations in the reference genome. An AB/WIK cross from another mutant (*m1193*) under characterization was sequenced at lower coverage (fourfold coverage) in order to increase the collection of zebrafish SNPs. The sequence reads from the *m1045* analysis were aligned to the wild type Tü reference genome (DanRer7) (http://www.sanger.ac.uk/Projects/D_rerio/) using the Burrows-Wheeler Aligner program, which allows efficient alignment of short sequencing reads against a large reference sequence, taking into account mismatches and gaps [10]. The sequence reads were visualized with the integrative Genomics Viewer, a lightweight visualization tool that enables intuitive real-time exploration of large-scale genomic data sets on a standard desktop computer [11] (see for example Figure 4A–B). Using the mpileup command on the SAMtools software [12] (see Material and Methods), 11 million single nucleotide polymorphisms (SNP) were found all along the chromosomes, with an average frequency of 1 SNP per 130 base pairs. In almost all chromosomal regions, most of the SNPs were heterozygous as the mutant embryos can carry the WIK and/or the AB alleles (Figure 4A). In contrast, the chromosomal region carrying the *m1045* mutation should display high SNP homozygosity as all sequences of this region are derived from the original mutagenized AB male. Indeed, the probability of having a crossing over between the AB and the WIK allele is near zero in this region. To locate the region displaying identical reads as shown in Figure 3B, an algorithm was developed that calculates for each SNP position a homozygosity score in a sliding 5000 SNP window corresponding to a mean size of 0.6 to 0.7 Mb (see Material and Methods). This homozygosity score should approach the value of 1 only in the region spanning the causal mutation. The homozygosity score was plotted against its respective position on each chromosome (Figures 4C, 4D and S1). Visual analysis of the 25 graphs identified the largest region of homozygosity on chromosome 5 (Figure 3D). In contrast, none of the other 24 chromosomes displayed such large regions (see for example Figure 3C for chromosome 1 and Figure S1 for the other chromosomes). Surprisingly, three smaller regions on chromosome 5 also showed homozygosity scores close to 1 (see regions A, B and C on Figure 4D). As the analysis of the homozygosity score for the other mutant (*m1193*), used as a reference, did not highlight these three regions (Figure 4D), we can reasonably infer that these three regions are genetically linked to the *m1045* mutation. One hypothesis is that these regions are misplaced on the current DanRer7 assembly and therefore should not be excluded as potential locations for the *m1045* mutation. Based on a cut-off value of 0.98 for the homozygosity score, four genomic regions were selected as potential candidate regions on chromosome 5: a 1.8 Mb region (A: 14627205–16413005), a 1.0 Mb region (B: 18026787–18997981), a 1.8 Mb region (C: 23570838–25366321) and a 3.2 Mb region (D: 50765827–53947430). In conclusion, this method allowed us to map the *m1045* mutation to a total of 7.8 Mb on chromosome 5.

### Identification of a nonsense mutation in the *snapc4* gene as the causal mutation in the *m1045* line

**Figure 5:**
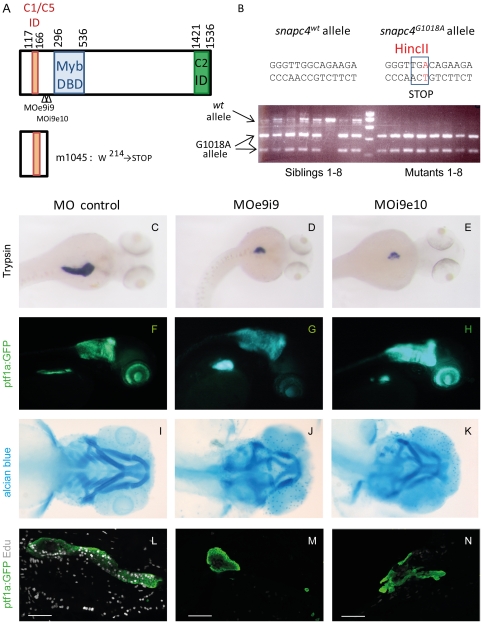
A nonsense mutation in the ORF of *snapc4* is the *m1045* causal mutation. A) Schematic scheme of the zebrafish Snapc4 protein. The conserved Myb DNA binding domain and the interacting domains for Snapc1/snapc5 and Snapc2 as described for the human *SNAPC4* ([16], [17] are indicated by boxes. The m1045 mutation generates a premature stop codon in the zebrafish *snapc4* cDNA resulting in a truncated protein of 213 aa. The positions targeted by the MOe9i9 and MOi9e10 *snapc4* morpholinos are indicated with arrowheads. B) Genotyping of *m1045* mutant and unaffected sibling embryos by RFLP analysis. C–N) Phenotype of embryos injected with 8 ng of control morpholino, 8 ng of MOe9i9 or 6 ng of MOi9e10 *snapc4* morpholinos. C–E) Dorsal view of WISH performed with a trypsin probe of 3.5 dpf larvae: 84% of the control morphants display a complete extension of the pancreatic exocrine tail (n = 19) while 52% of MOe9i9 morphants show a drastic reduction of the pancreatic exocrine tail and 48% no extension at all (n = 19) and 9% of MOi9e10 morphants show a drastic reduction of the pancreatic exocrine tail and 91% no extension at all (n = 23). F–H) Lateral view of 3.5 dpf Ptf1∶GFP larvae: 95% of the control morphants display a complete extension of the pancreatic exocrine tail (n = 25) while 13% of MOe9i9 morphants show a drastic reduction of the pancreatic exocrine tail and 87% no extension at all (n = 83) and 24% of MOi9e10 morphants show a drastic reduction of the pancreatic exocrine tail and 62% no extension at all (n = 55). I–K) Alcian blue staining of 4 dpf embryos: MOe9i9 and MOi9e10 morphants display a reduction and malformation of the hyoid concomitant with the absence of the branchial arches 3 to 7. L–N) Confocal projections of the pancreatic region of embryos injected with Edu at 95 hpf and fixed at 96 hpf. All the control morphants show a high Edu incorporation rate (n = 15). 95% of the MOe9i9 morphants did not incorporate any Edu at all (n = 19) and 43% of the MOi9e10 morphants display no incorporation at all and 21% a considerably reduced incorporation rate (n = 14). Scale bars : 50 µm.

In order to identify the causal *m1045* mutation within these four intervals, we next searched for all transcripts present in these regions by exporting from the UCSC genome informatics website (http://genome.ucsc.edu) [13] the RefseqGenes [14] and the Ensembl transcripts [15]. In total, 72 refseq genes and 224 ensembl transcripts were localized in these regions. From the 47,071 observed SNPs in these regions, 195 were located within coding regions and create an amino acid change. Among these, 31 were private to the *m1045* genome, which means that they were not detected as sequence variants either in the Tü or in the *m1193* genome (see Supplemental Table S1). From these, only one was a nonsense mutation at position 52.8 Mb on chromosome 5 in the transcripts ENSDART00000097473 and ENSDART00000141424, coding for a partial Snapc4 (small nuclear RNA activating complex, polypeptide 4) protein. Homology searches allowed us to identify the full length *snapc4* cDNA (Genbank accession number, JQ434101) (see Material and Methods). The Snapc4 protein is 1,557 amino acids long and presents respectively 27% and 32% identity over its entire peptidic sequence with the human and chicken ortholog (Figure S2). The SNAPC1/SNAPC5 interacting domain, the Myb DNA binding domain and the SNAPC2 interacting domain, as described in humans [16], [17], are well conserved in the zebrafish Snapc4 protein but not the Oct-1 interacting domain (see Figures 5A and S2). The mutant *m1045* allele contains a G to A base substitution at position 1018 in exon 10 of the *snapc4* transcript, leading to a premature stop codon at position 214 (Figure 5A). Consequently, about 80% of the protein is missing in the mutant strain and notably the Myb DNA binding domain, essential for the function of the human protein [17].

To determine whether the G1018A nonsense mutation in the *snapc4* gene was the causative mutation, we first verified that this G1018A substitution was found in a homozygous state in mutant embryos but not in the unaffected siblings. As the point mutation creates a fortuitous HincII restriction site, the mutant allele was easily identified by restriction analysis of the PCR product spanning this region. We genotyped 21 mutants and siblings by this method and found that all mutant embryos were *SNAPC4* ^G1018A^ homozygous while the siblings were wt or heterozygous (Figures 5B and S3). This indicated that either *snapc4* ^G1018A^ was the causative mutation or it was closely linked to *m1045*.

To definitively prove that the *m1045* phenotype was caused by the *snapc4* ^G1018A^ nonsense mutation, two splice-blocking morpholinos were injected into the embryos to knockdown *snapc4* gene function. Injection of 8 ng of MOe9i9 or 6 ng of MOi9e10 interfered with the correct splicing of the transcript (see Figure S4) and led to a reduction in the expression of the *trypsin* and *ptf1a* exocrine markers at 3.5 dpf (Figure 5 C–H). In contrast, the injection of an equivalent quantity of control morpholino did not have any effect on the exocrine tissue (Figure 5 C,F). Alcian blue staining of the morphants also revealed defects in the formation of the branchial arches with a reduction and malformation of the hyoid concomitant with the absence of branchial arches 3 to 7 (Figure 5I–K), as observed in the *m1045* mutant (for comparison, see Figure 1H). Finally, cell proliferation was completely abolished in the pancreatic exocrine tissue of both morphants (Figure 5L–N) as well as in all tissues of the larvae at 4 dpf (data not shown).

As the injection of two different *snapc4* morpholinos phenocopies the *m1045* mutant, we conclude that the *snapc4^G1018A^* is the causal mutation responsible for the pancreatic hypoplasia of the *m1045* mutant larvae.

### Expression profile of Snapc4 protein

**Figure 6:**
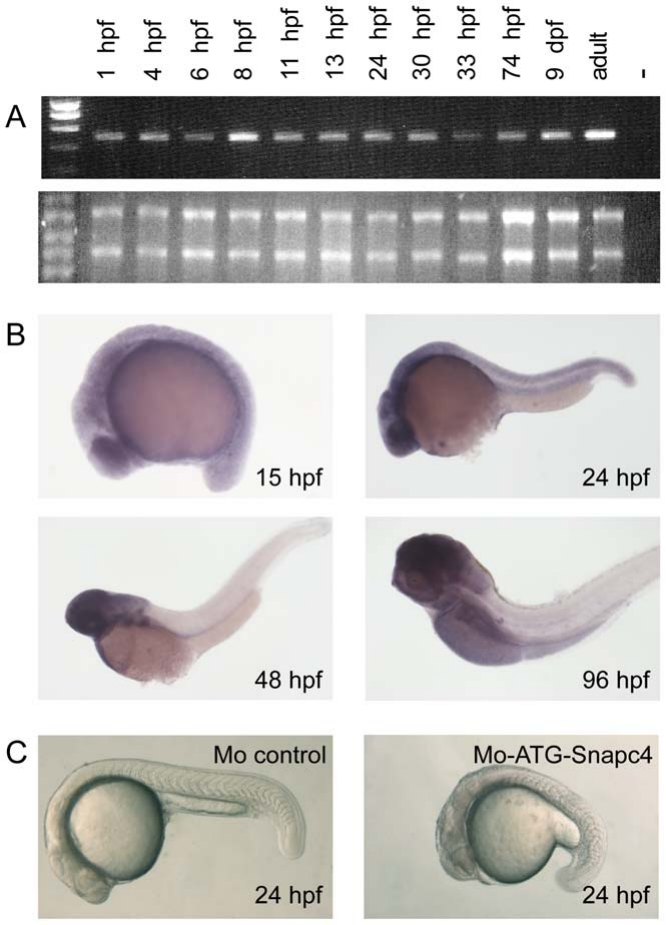
A: Time course analysis of zebrafish *snapc4* expression by semi-quantitative RT-PCR. A control without addition of RNA (lane -) was included as a negative control. 3 µg of total RNA extracted at each time point were loaded on a denaturating agarose gel to check the quality and the quantity of the RNA used for each RT-PCR. **B: Expression profile of the** ***snapc4*** **transcripts** performed by WISH at 15 hpf (12S), 24 hpf, 3 dpf and 4 dpf. **C: Translation-blocking snapc4 morpholino leads to growth retardation before 24 hpf.** Bright-field images of 24 hpf embryos injected with 8 ng of control morpholino or of MO_ATG_-snapc4 : 75% of the embryos injected with MO_ATG_-snapc4 show severe growth retardation compared to the control morphant.

The temporal and spatial expression profile of the zebrafish *snapc4* gene was analysed by a reverse transcriptase-PCR (RT-PCR) assay and whole-mount in situ (WISH). *snapc4* transcripts are maternally provided since we could detect them before the onset of zygotic transcription, which occurs around 3.0 hpf [18] (Figure 6A). The *snapc4* transcripts level remained quite constant at all stages analysed. WISH showed a ubiquitous expression of *snapc4* during the first day of development (Figure 6B). At later stages, *snapc4* expression became mostly restricted to the gastrointestinal tract, the eyes, the jaw and the brain.

### A translation-blocking morpholino targeting the maternal *snapc4* transcript severely affects embryonic development at early stages

The expression of *snapc4* at an early stage contrasted with the phenotype of the *m1045* mutant or the *snapc4* splice-blocking morphants where defects were visible only after 3 dpf. A possible explanation is that *snapc4* is not essential for cell division during the early developmental stages in zebrafish. Alternatively, maternal deposits of *snapc4* RNA and/or proteins might be sufficient for development to proceed through embryogenesis until the maternal contribution is exhausted. To test these hypotheses, we injected a translation-blocking morpholino, MO_ATG_-Snapc4, that targets both maternal and zygotic *snapc4* mRNA. Injecting 8 ng of MO_ATG_-Snapc4 severely impeded embryonic development as 25% of the embryos showed developmental arrest and died before 24 hpf and the others showed a severe reduction in growth (Figure 6C) (n = 80). This result supports the idea that the maternal *snapc4* transcripts are able to complement at early stages for the loss of zygotic Snapc4 protein in the *m1045* mutant or in the *snapc4* splice-blocking morphants.

## Discussion

WGS of mutant organisms displaying specific defects is a very promising approach for determining the genetic determinants of a plethora of biological processes. While this approach was recently shown to be feasible for *C. elegans* and *Drosophila* [5], [6], it had not yet been described for zebrafish, whose genome is approximately tenfold larger. By identifying by WGS a nonsense mutation in the *snapc4* gene that causes hypoplasia of the exocrine pancreas, we have demonstrated that this strategy can also be applied in zebrafish to identify rapidly mutations producing phenotypes of interest.

In mice, WGS has also been successfully applied for identifying the causative mutation responsible for renal failure [19]. However, in that study, WGS was coupled with genetic mapping by bulk segregation analysis to determine which of the many sequence variants identified in the genome were associated with the phenotype. Here, prior knowledge of linkage was not necessary as our algorithm calculating a SNP homozygosity score along the chromosomes enabled us to define the interval carrying the mutation. The search for sequence variants in the mutants was therefore restricted to this interval.

Mapping by WGS offers many unique advantages. This approach requires a much lower number of mutants than the traditional SSLPs fine mapping strategy. Indeed, we were able to map the mutation with a pool of 50 mutants. The sequence run and analysis takes a few weeks, which is a substantial time saving compared to several months needed for finding the critical region using polymorphic SSLP markers. Nowadays it can be done with minimal costs and, furthermore, our method does not require the sequencing of the wt siblings. In addition, we have demonstrated here that it is not necessary to perform a deep sequencing of the whole zebrafish genome. An eightfold coverage of the zebrafish genome (13 Gb of sequence) was sufficient to define the interval carrying the mutation. Moreover, with this coverage, nearly all transcribed regions were sequenced (98% of the exons showed an average coverage of at least 5 times (see Figure S5)).

The chromosomal region containing the causal mutation was identified via the analysis of SNP homozygosity. In order to enrich the collection of SNPs, the sequence of another AB/WIK cross performed with the mutant *m1193* was determined at lower coverage (fourfold coverage). By compiling the sequences of any mutants performed in the future, we will be able to establish an exhaustive list of all possible SNPs in zebrafish, which will surely refine the mapping analysis. The interval carrying the mutation was located using an algorithm that calculates the SNP homozygosity score along all chromosomes. This score should theoretically reach the value of 1 near the causal mutation because that region derives exclusively from the original mutagenized male. For the *m1045* mutant, only chromosome 5 displayed a large region with a homozygosity score of 1, allowing us to map the causal mutation between positions 50.7 to 53.9 Mb on chromosome 5. Surprisingly, we identified three smaller regions around 15, 18 and 24 Mb on chromosome 5 that also showed a homozygosity score of 1 (see Figure 4D). One explanation could be that these three regions are misplaced on chromosome 5 in the current DanRer7 build and should be located contiguous to the 51 to 54 Mb region. We noticed that the two first peaks were positioned around 12 and 14 Mb on the previous DanRer6 build indicating that chromosome 5 assembly is not yet fully completed. Further experiments will be required to verify this hypothesis.

While misassembled regions can cause huge difficulties for the traditional SSLP mapping strategies, they will not interfere with the identification of the causal mutation by WGS if they are sufficiently large to be detected by our “homozygosity score algorithm". Indeed, sequence variations in these putative misplaced regions should also be listed and, in the case of the *m1045* mutant, they did not contain any nonsense mutations.

WGS not only allows identification of the chromosomal region carrying the causal mutation but also permits the analysis of the sequence variations in this interval to pinpoint the putative molecular lesion. Based on more than 100 mutations already identified in zebrafish forward screens, about half of them (46%) introduce a nonsense codon, 15% alter a splice site and 34% correspond to missense mutations [20]. Nonsense mutations and mutations affecting the splicing are quite easy to detect. In contrast, identifying missense mutations is more challenging as it can be difficult to distinguish the causal mutation from natural polymorphisms present in the fish. The search for sequence variations located in conserved domains of proteins can be used as a hint for detecting deleterious missense mutations. However, if all these steps do not lead to the identification of the causal mutation, it can be unambiguously determined by performing WGS of the original mutagenized male from which the mutant line originates. Indeed, sequence comparison of the genome sequences of the mutagenized male and the mutants will allow subtraction of all nucleotide variants that are common to this particular strain. In contrast, the causal mutation will not be detected in the sequence obtained from WGS of genomic DNA extracted from the whole mutagenized male fish. Indeed, ENU mutagenesis of the male is highly mosaic and therefore the ENU mutation that gave rise to a particular mutant is only present in the DNA of a small subset of germ line cells. As the WGS is performed on the genomic DNA extracted from the whole mutagenized male, the chance of detecting the causal mutation in the sequence reads is almost zero. This underlines the importance of keeping genomic DNA from all mutagenized males as well as documenting the pedigree of the mutants.

In this study, we identified a zebrafish *snapc4* mutant in which cell proliferation is completely abolished at 3 dpf leading to hypoplasia of tissues that undergo a dramatic growth at this stage, such as exocrine pancreas, eyes and branchial arches. The snRNA-activating protein complex SNAPc is a multi-subunit complex composed of at least five subunits: SNAPC1 (also known as SNAP43 [21]), SNAPC2 (SNAP45 [22]), SNAPC3 (SNAP50 [23]), SNAPC4 (SNAP190 [24]) and SNAPC5 (SNAP19 [25]). SNAPC4 forms the backbone of the complex and binds three of the four remaining subunits (SNAPC1, SNAPC2 and SNAPC5) while SNAPC3 joins the complex through contact with SNAPC1. The SNAPc complex is essential for the transcription of all snRNA genes, including U1, U2, U4, U5 and U6 spliceosome snRNAs [26]. It binds to the proximal sequence element (PSE) which is found in all human snRNA promoters (reviewed by [27]). DNA binding by SNAPc requires both SNAPC4 and SNAPC3 which directly bind to DNA via their Myb and zinc-finger DNA binding domains, respectively [24], [28]. As the SNAPC4^G1018A^ allele, as found in the *m1045* mutant, gives rise to a protein of 213 amino acids that does not contain the Myb DNA binding domain (see Figure 5A), this strongly suggests that the SNAPC4^G1018A^ is a null mutation.

Obviously, interfering with snRNAs formation and notably with the spliceosomes would have a detrimental consequence for the development of any organisms. For example, blockage of U2 snRNA function induces early developmental arrest in zebrafish [29]. In human cell lines, *SNAPC4* downregulation resulted in an accumulation of cells with a G_0_/G_1_ DNA content and a concomitant decrease of cells in S and G2/M phases [30]. In the *m1045* mutant, the cell proliferation defect does not occur before 3 dpf. A possible explanation is that maternal deposits of RNA and/or protein might be sufficient for development to proceed through embryogenesis until the maternal contribution is exhausted. Consistent with the maternal-store depletion hypothesis, RT-PCR revealed that *snapc4* is maternally expressed in zebrafish (Figure 6A). Moreover, the two splice-blocking morpholinos (MOe9i9 and MOi9e10) that target only zygotic *snapc4* transcripts, led to cell proliferation defects only at a late stage. In contrast, the embryos injected with a translation-blocking morpholino (MO_ATG_-Snapc4), that in addition targets the maternal transcript, display earlier defects with embryos showing either developmental arrest before 24 hpf or a drastic growth retardation. All these data support the idea that the maternal *snapc4* transcripts are able to complement at early stages for the loss of zygotic Snapc4 protein in the *m1045* mutant.

## Materials and Methods

### Zebrafish strains and ENU mutagenesis

Embryos and adult fish were raised and maintained under standard laboratory conditions. All animal work has been conducted according to national guidelines and all animal experiments described herein were approved by the University of Liege (protocol number 371). The transgenic *Tg(Ptf1∶GFP)* line was kindly provided by Steven Leach [7]. ENU mutagenesis was performed as previously described [31]. The newly isolated alleles used here are *m1045* carrying a *snapc4^G1018A^* nonsense mutation and *m1193* used as reference. AB (ZL1) and WIK (ZL84) wt strains were obtained from the Zebrafish International Resources Center (ZIRC).

### DNA preparation and Illumina whole-genome sequencing

Fish heterozygous for the *m1045* alleles in AB strain were crossed to the WIK strain and subsequently *m1045* (AB)/+ (WIK) females were crossed with *m1045* (AB)/+ (AB) males to generate *m1045* mutant embryos and their siblings (Figure 3). Genomic DNA from 50 pooled homozygous mutants was prepared using Maxwell® 16 Tissue DNA Purification Kit (Promega) and was quantified using PicoGreen® dsDNA Quantitation kit (Molecular Probes). Genomic shotgun library was prepared according to the manufacturer's protocol (Illumina, Paired-end Sequencing Sample Preparation guide). Briefly, 1 µg of genomic DNA was fragmented using the bioruptor NGS (8 cycles 15 s on, 90 s off) (Diagenode, Belgium), ends-repaired and ligated with genomic adapters after addition of a 3′-A. The fragments were size-selected on agarose gel (∼400 bp) and submitted to PCR amplification. Quantification and quality control of DNA was performed on a 2100 Bionalyser using the Agilent High Sensitivity DNA Kit (Agilent). The paired end library was sequenced on 3 lanes for 2 times 76 cycles on an Illumina GAIIx sequencer using SBS sequencing kits V4.0 generating 12.6 Gb of sequence. Base calling was done with SCS 2.8/RTA 1.8. The DNA obtained from a pool of 50 mutant embryos originating from a cross between the WIK strain and the *m1193* mutant AB line was prepared following the same strategy and protocol and sequenced on one lane to generate 6.3 Gb paired-end 76-mer sequence representing a 4× coverage of *m1193* genome. This sequence was used together with the Tü reference sequence to generate a genome wide SNPs collection (see below).

### Sequence reads mapping and SNP detection

The paired end reads were mapped to the *Danio rerio* Zv9 assembly (GCA000002035.2) with the open source Burrows-Wheeler Aligner (BWA) short read mapper [10] using the default parameters. This program allows to efficiently align short sequencing reads against a large reference sequence, allowing mismatches and gaps [10]. BWA outputs alignment in a standard SAM (Sequence Alignment/Map) format which can be read by the Integrative Genomics Viewer (IGV) and the Samtools software. IGV enables real-time exploration of large data sets over all resolution scales, while consuming minimal resources on the client computer. The identification of the SNPs has been performed with the mpileup command of SAMtools (*options*: -C50 -D -S) [12] based on the comparison with the Zv9/danRer7 reference sequences (Tü strain) and the sequences obtained from the *m1045* and *m1193* mutants. The option -C50 allows to reduce the effect of reads with excessive mismatches (http://samtools.sourceforge.net/mpileup.shtml) by filtering out reads with mapping quality below 50. The -D and -S options enable to keep per-sample read depth and strand bias, options which are preferred if there are multiple samples. These parameters were selected to have a high sensitivity to maximize the chance of keeping the causal mutation in the list of SNPs.

For the first step of the SNP analysis, which aims to determine the interval carrying the m1045 mutation, the genotype calls produced by mpileup for the *m1045* mutants were used to evaluate SNP homozygosity. Homozygous SNP positions were given a score of 1 and heterozygous positions a score of 0. Genotype calls were filtered for a minimum Genotype Quality score (GQ) of 5 to gain in specificity for this first mapping step. The genome was scanned for regions showing a high homozygosity by calculating a mean score in all windows of 5000 SNP sites. For each SNP, all windows encompassing the SNPs were evaluated and the highest mean score was retained.

For the second step of the SNP analysis, which aims to identify the causal mutation, all the SNPs found in the regions with a SNP homozygosity score of at least 0.98 were listed and SNPs also found in the m1193 and Tü strains were filtered out. The remaining SNPs were then annotated using SQL scripts interrogating the DanRer7 mySQL annotation database of UCSC (genome-mysql.cse.ucsc.edu). The SNPs were classified as noncoding, altering splice-acceptor or splice-donor sites, synonymous, missense or nonsense.

### RFLP analysis of the *snapc4* ^G1018A^ allele

To genotype embryos for the presence of the *snapc4^G1018A^* allele, PCR with the primers O202 (TGTGTTTGACCCCAAAAGTCTT) and O203 (GGACAACAATATCTCCAGTTGAAAA) were performed on genomic DNA. HincII restriction of the 683 bp amplicons generated a restriction fragment length polymorphism (RFLP) of 435 bp and 248 bp for the *snapc4* ^G1018A^ allele and of 683 bp for the *snapc4* ^wt^ allele.

### Identification of the full length *snapc4* cDNA

Incomplete snapc4 transcripts, ENSDART00000141424, ENSDART00000097473 and ENSDART00000132503, coding for partial Snapc4 proteins (627, 393 and 85 aa, respectively) were found on Ensembl (http://www.ensembl.org/Danio_rerio). These transcripts did not contain the coding region for the C-terminal part of the protein and notably the Snapc2 interacting domain. *In silico* homology searches using the human SNAPC2 interacting domain identified orthologous zebrafish genomic sequences 20 kb apart from the ensembl transcripts. Blast (http://blast.ncbi.nlm.nih.gov/Blast.cgi) searches using this zebrafish genomic sequence identified a series of expressed sequence tags (EST) that, when assembled, generates a 2 kb fragment coding for the C-terminal part of Snapc4. To link the C-terminal and the N-terminal parts of the snapc4 transcript, PCR was performed on 24 hpf cDNAs using primers O244 (CCGGCTTAGGCTCCGATTCTTCAGA) and O245 (GCACACTTGGCGATTTGGAGACTAC) located on both sides and the PCR fragments were sequenced. By this way, we obtained a full length cDNA sequence of 5371 pb, composed of 27 exons. The *snapc4* sequence was deposited in Genbank (accession number: JQ434101).

### Whole-mount in situ hybridization (WISH)

Whole-mount in situ hybridizations were performed as described previously [32] with the trypsin riboprobe [33] and a 920 bases snapc4 riboprobe. The SNAPC4 clone, purchased from Imagenes (IMAGp998d046487Q) (Germany), was provided in the pME18S-FL3 vector containing a SNAPC4 EST (AW134394). The cDNA insert was amplified by PCR using oligonucleotides developed to the pME18S-FL3 vector (BP628 : TGTACGGAAGTGTTACTTCTGCTC and BP629, containing a T3 promoter : GGATCCATTAACCCTCACTAAAGGGAAGGCCGCGACCTGCAGCTC). The PCR fragment was subsequently digested with BamH1 and the snapc4 riboprobe synthesized using the T3 polymerase.

### Alcian Blue staining

Cartilage was stained with Alcian Blue 8 GX (Sigma®) as described by [34]. Briefly, four days old embryos were fixed in PFA 4% for 2 h at room temperature or ON at 4°C, rinsed with PBST and finally stained overnight with 10 mM Mg Cl2/80% ethanol/0.04% Alcian Blue solution. Embryos were rinsed with 80% ethanol/10 mM MgCl2. Pigments were bleached in H2O2 3%/KOH 0,5% for 1 h and rinse with 25% glycerol/0,1% KOH for 1 h and mounted in 50%Glycerol/0,1% KOH.

### Haematoxylin/eosin staining

PFA fixed embryos were dehydrated, embedded into JB-4 plastic resin (Polysciences, Inc.), sectioned at 4 µm on a Leica microtome and stained in haematoxylin and eosin.

### Proliferation Analysis

EdU incorporation and detection were performed as described previously [35] using the Click-IT 555 or 647 kit (Invitrogen C10338 or C10085) according to manufacturer's instructions. Briefly, tricaine-anesthetized larvae were injected into the yolk with approximately 5 nL EdU solution (1 µM/2% DMSO/0.1% phenol red), let recovered for 1 hour at 28°C and then immediately fixed overnight in PFA 2%/Pipes 0.1 M/MgSO_4_ 1 mM/EGTA 2 mM at pH 7. The next day, samples were then washed 3 times with PBS+0.3% Triton X-100, de-yolked, treated for 40 min in PBS+1% Triton X-100, rinsed once with ddH20, and then reacted with 250 µL fresh click-iT reaction cocktail for 20 min. To increase the sensitivity of GFP detection, an immunohistochemistry was performed subsequently as described in [36] using chicken anti-GFP (1/500, Aves Labs).

Fluorescent images were acquired with a Leica SP2 confocal microscopes and Maximum Intensity Projections were performed with the Imaris software (Bitplane).

### Morpholino design and injection

Morpholino oligonucleotides (MO) were synthesized by Gene Tools (Corvalis, OR). Each MO was resuspended in Danieau's solution at the stock concentration of 1 mM or 2 mM. For injection, this stock solution was diluted as specified in Danieau's solution and 1000 pl were injected into the yolk of one-cell stage ptf1∶GFP embryos. To check the injection efficiency, rhodamine dextran was added at 0,5% in the injected solutions. To block the expression of *snapc4*, we used two splicing morpholinos : 8 ng of MOe9i9 (CATGCTGTCTTAATACGTACATCTT), targeting the junction between the ninth exon and the ninth intron or 6 ng MOi9e10 (TCCCCTGAAAGACATAACACAACGT), targeting the junction between the ninth intron and the tenth exon and 8 ng of a translation-blocking morpholino MO_ATG_-Snapc4 (TCCAAAAATGGCATCTGACGACTTA) spanning the ATG start site. The standard control MO (CCTCTTACCTCAGTTACAATTTATA) designed by Gene Tools was used as negative control. To control the morpholino efficiency of the splicing-blocking morpholinos, total RNA of morphants were extracted at 30 hpf as described below. RT-PCR was performed on 1 µg of total RNA. The primers used for PCR amplification were O219 (GCTCATTGAAAATCAACAGCAGCA) and O220 (CTGACGCAAACCTTCAAAATCGATA). Amplified cDNAs were analyzed by gel electrophoresis and sequencing.

### RNA extraction, cDNA synthesis and RT-PCR of the *snapc4* transcript

Total RNA of whole embryos/fishes at different stages were isolated using Trizol™ Reagent (Life technologies) as described previously ([37]. Total RNA (5 µg) were then reverse transcribed with Superscript™ reverse transcriptase (Superscript™ first strand synthesis system for RT-PCR, Invitrogen) and random hexamers as primers. Semi-quantitative PCR amplification of the *snapc4* transcript was performed using the primers O219 and O220.

### Footnotes

The sequencing and the analysis of the sequences as described in this study can be performed upon demand by the GIGA-Geno-Transcriptomics Technology Platform (http://www.giga.ulg.ac.be/jcms/prod_206410/services).

## Supporting Information

Figure S1 **Analysis of the homozygosity scores for** ***m1045*** **and m1193 on all chromosomes.** SNPs homozygosity score for *m1045* (in red) and for *m1193* (in green) plotted against their respective position for the 25 chromosomes.
(TIF)

Figure S2 **Alignment of vertebrate** ***SNAPC4*** **peptidic sequences.** Residues identical in all proteins are shaded in yellow and those conserved in just some of them are shaded in blue. The interacting domains and the Myb DNA binding domain as described for the human SNAPC4 ([16], [17] are indicated by boxes. Note that the Oct-1 interacting domain is not conserved in zebrafish. Dr-Snapc4 (JQ434101) Hs-SNAPC4 (OTTHUMP00000022583), Gg-SNAPC4 (Xp415416) and Mm-SNAPC4 (OTTMUSP0000013728). Dr: Danio rerio, Hs : Homo sapiens, Gg : Gallus gallus, Mm : Mouse musculus.
(PDF)

Figure S3 **Genotyping of** ***m1045*** **mutant and unaffected sibling embryos by RFLP analysis.**
(TIF)

Figure S4 **Control of the morpholino efficiency of the splicing-blocking morpholinos.** RT-PCR analysis of total RNA extracted from 30 hpf morphants show that the snapc4 mRNA is truncated in the Moe9i9 and Moi9e10 morphants.
(TIF)

Figure S5 **Average sequence coverage of the exons of the 13761 refseq genes.**
(TIF)

Table S1 **List of the SNPs within the A, B, C and D homozygosity regions on chromosome 5 that create an aminoacid change and are specific to the** ***m1045*** **genome.** The position of the SNP on the chromosome 5 is indicated, the codon affected and the type of substitutions: the column “aa reference" corresponds to the aa found in the Tü genome while the column “aa m1045" correspond to the aa found in the m1045 genome. 30 SNPs create missense variations compared to the reference while only one SNP (underlined in yellow) creates a STOP codon in the transcripts ENSDART00000097473 and ENSDART00000141424, coding for the Snapc4 protein.
(PDF)
