# Supplementary figures and images for: Fast Homozygosity Mapping and Identification of a Zebrafish ENU-Induced Mutation by Whole-Genome Sequencing
Source: PLoS One. 2012 Apr 4;7(4):e34671. doi: 10.1371/journal.pone.0034671 (PMC3319596; doi:10.1371/journal.pone.0034671)

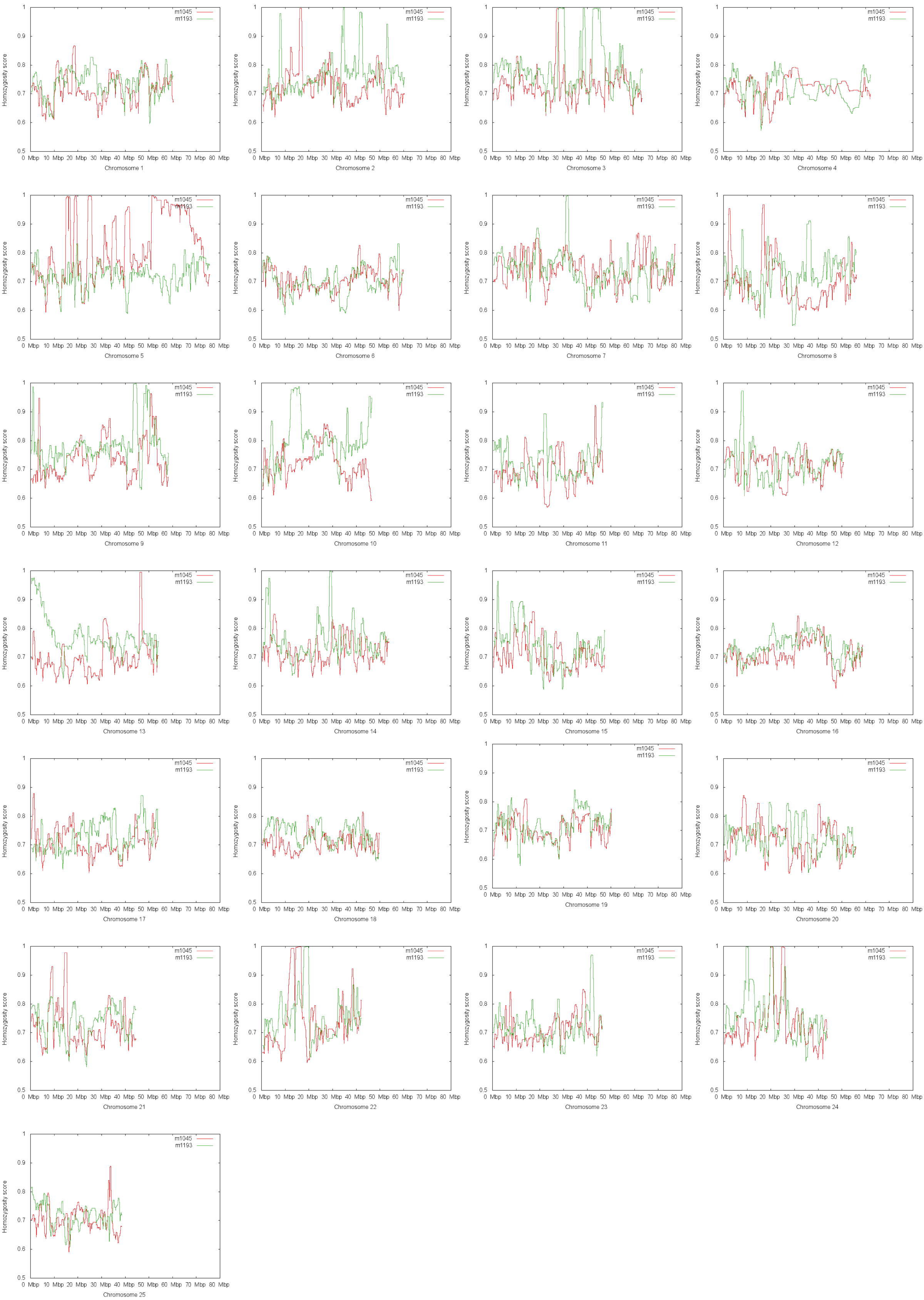

Supplement: Figure S1 — Analysis of the homozygosity scores for m1045 and m1193 on all chromosomes. SNPs homozygosity score for m1045 (in red) and for m1193 (in green) plotted against their respective position for the 25 chromosomes. (TIF) [file pone.0034671.s001.tif]

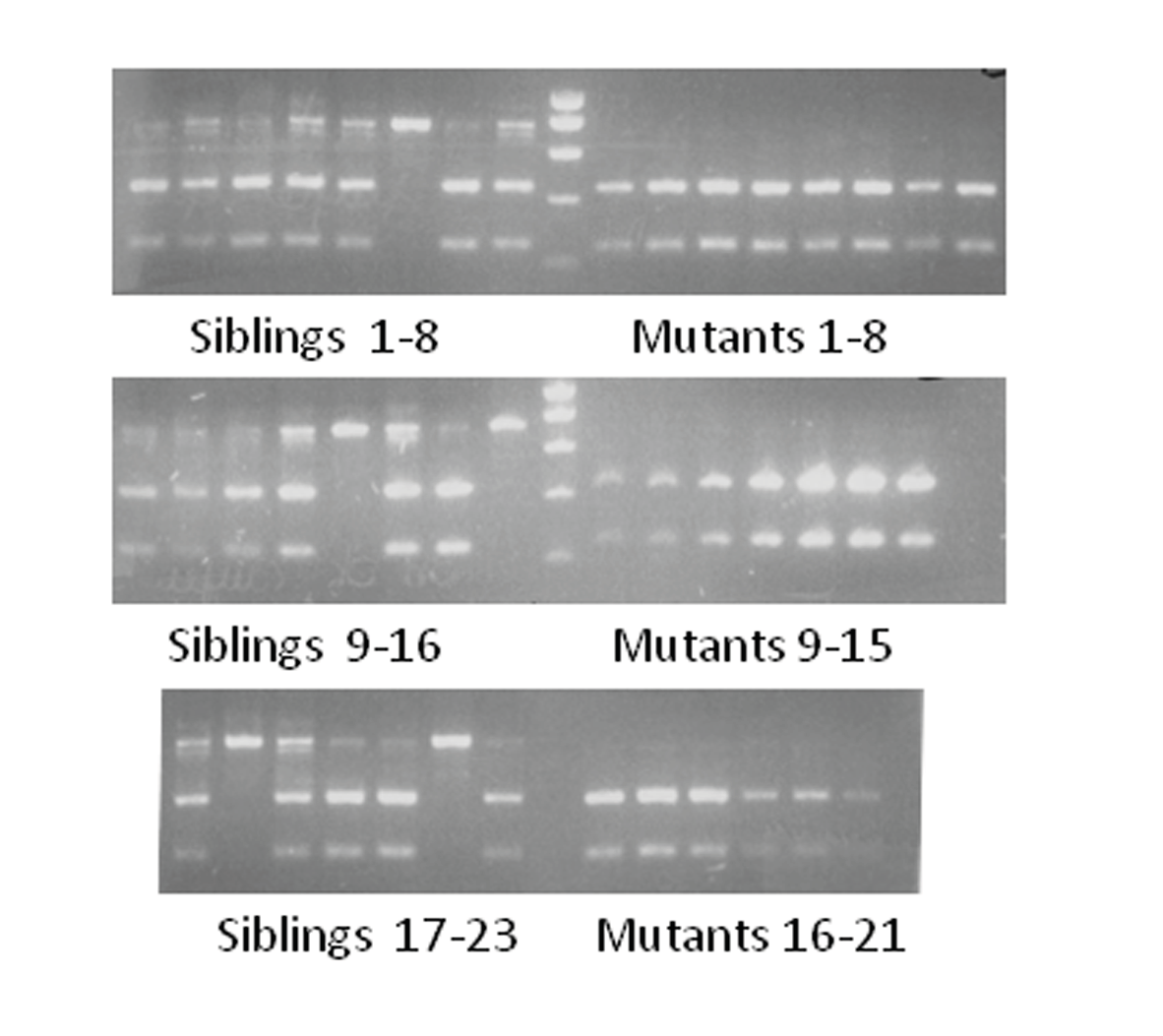

Supplement: Figure S3 — Genotyping of m1045 mutant and unaffected sibling embryos by RFLP analysis. (TIF) [file pone.0034671.s003.tif]

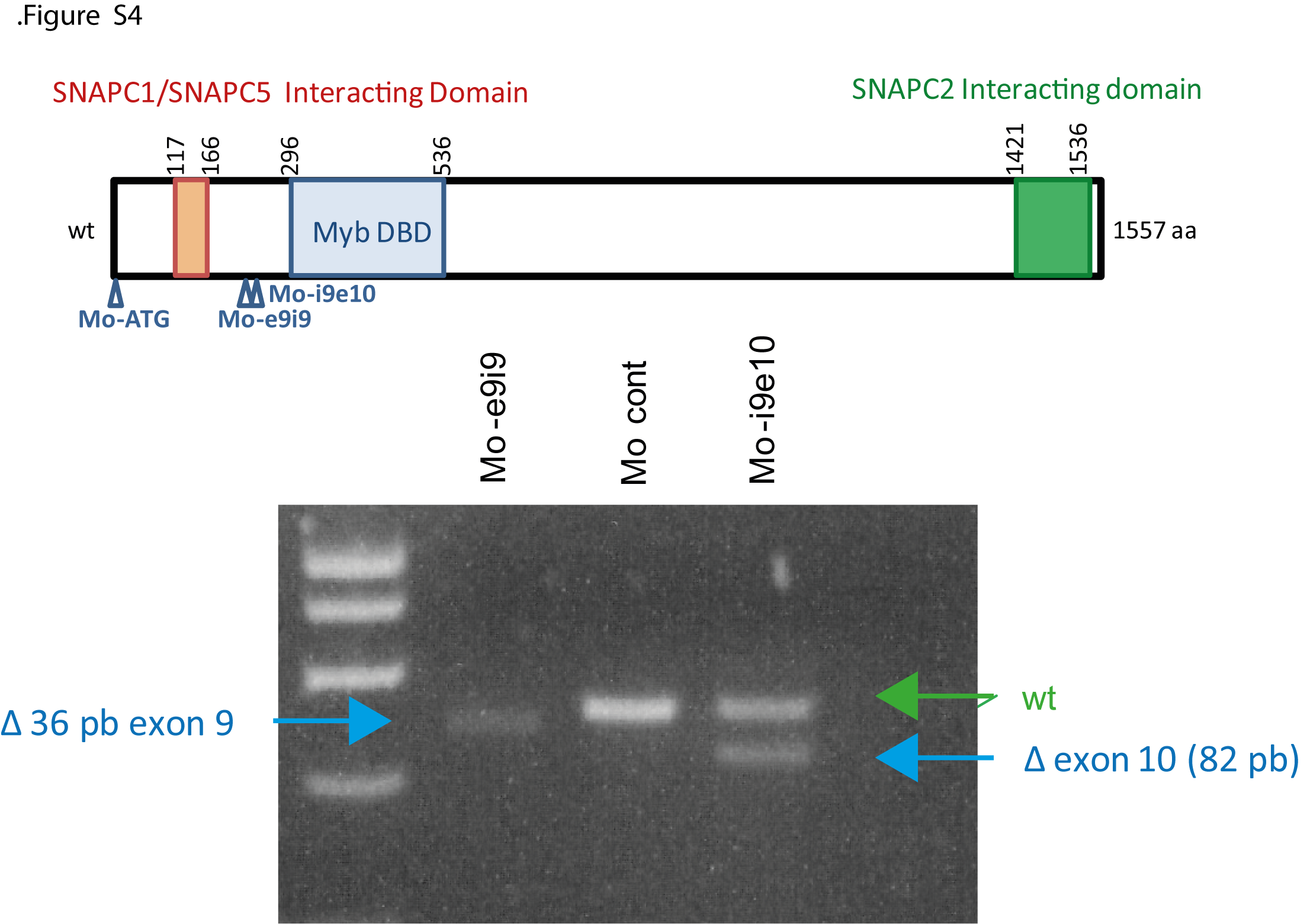

Supplement: Figure S4 — Control of the morpholino efficiency of the splicing-blocking morpholinos. RT-PCR analysis of total RNA extracted from 30 hpf morphants show that the snapc4 mRNA is truncated in the Moe9i9 and Moi9e10 morphants. (TIF) [file pone.0034671.s004.tif]

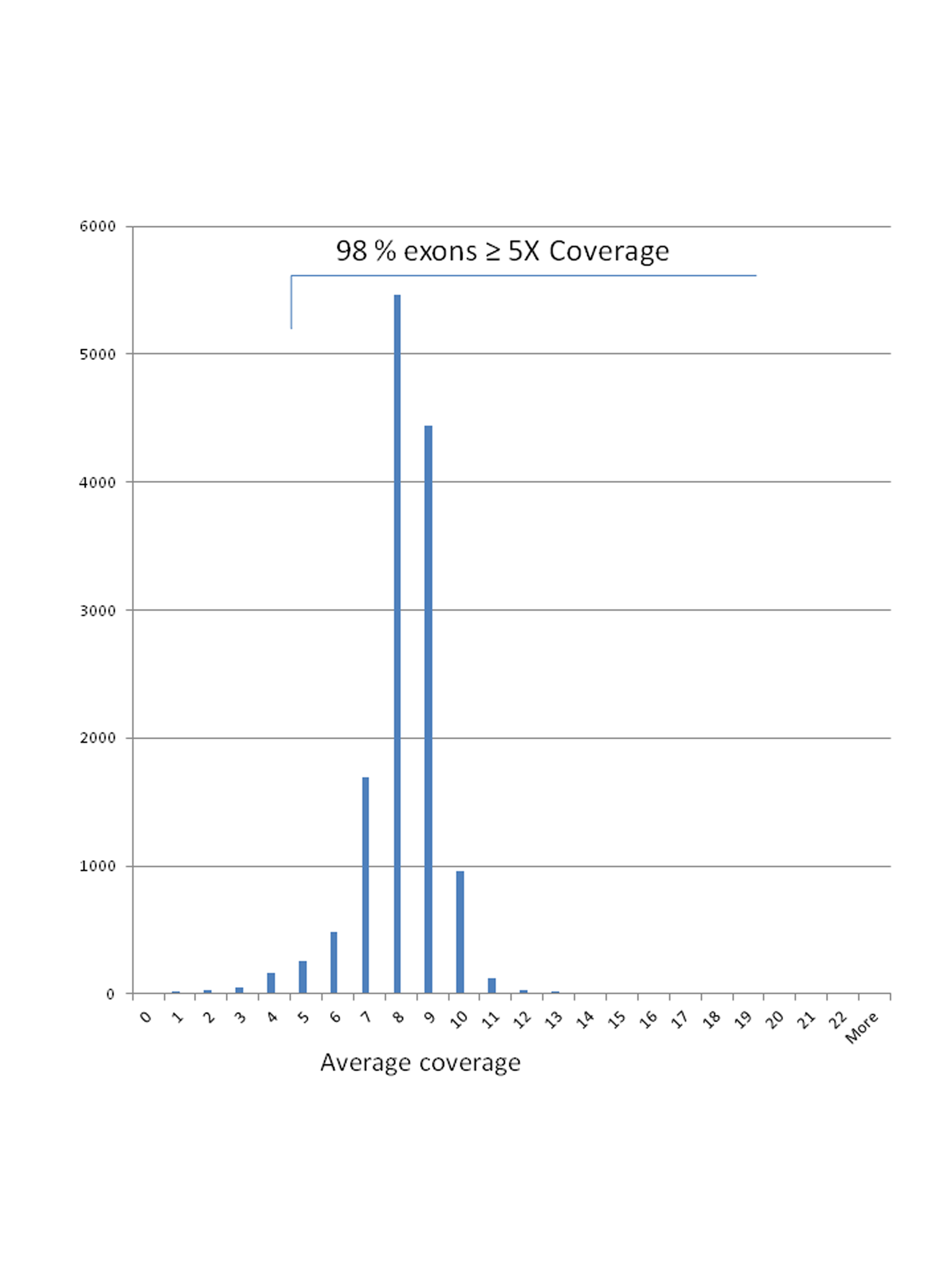

Supplement: Figure S5 — Average sequence coverage of the exons of the 13761 refseq genes. (TIF) [file pone.0034671.s005.tif]
