# Supplementary material for: Fast Homozygosity Mapping and Identification of a Zebrafish ENU-Induced Mutation by Whole-Genome Sequencing
Source: PLoS One. 2012 Apr 4;7(4):e34671. doi: 10.1371/journal.pone.0034671 (PMC3319596; doi:10.1371/journal.pone.0034671)
Supplement: Figure S2 — Alignment of vertebrate SNAPC4 peptidic sequences. Residues identical in all proteins are shaded in yellow and those conserved in just some of them are shaded in blue. The interacting domains and the Myb DNA binding domain as described for the human SNAPC4 ([16], [17] are indicated by boxes. Note that the Oct-1 interacting domain is not conserved in zebrafish. Dr-Snapc4 (JQ434101) Hs-SNAPC4 (OTTHUMP00000022583), Gg-SNAPC4 (Xp415416) and Mm-SNAPC4 (OTTMUSP0000013728). Dr: Danio rerio, Hs : Homo sapiens, Gg : Gallus gallus, Mm : Mouse musculus. (PDF) [file pone.0034671.s002.pdf]

```
       (1) 1        10        20        30        40        50        60        70        80        90        100
gg-SNAPC4  (1) MSRRDGPTGANLNAEREKIRREIEELERSLQLDVTSVDVAVSDSSLSSDDAEGDSSDAHAEMEVERE---------------------------------
mm-SNAPC4  (1) -MPRRQAEAMDIDAEREKITQEIQELERILYPGSTSVHFEVSESSLSSDSEADSLPDEDLETAGAPILE------------------------EEGSSES
hs-SNAPC4  (1) ---------MDVDAEREKITQEIKELERILDPGSSGSHVEISESSLESDSEADSLPSEDLDPADPPISE------------------------EERWGEA
dr-SNAPC4  (1) ------MASDDLQAQRDKIQREILALESTLGADSSIADQLSSDNSSDYESDDSGPTVERVERDDLETERLRIQREIEELENALGADAALENVLQDSDHDT
```

SNAPC1/SNAPC5 Interacting Domain

```
     (101) 101      110       120       130       140       150       160       170       180       190       200
gg-SNAPC4 (68) EDSSDDDLESSLLEDPETCLQMNYVYQEVIQEKIEEVELLIAQNKEQQKEILCELDGRKTTRTGDGRNLPSNIFLGHFMKPYFKDKTTGIGPPSNEDAKE
mm-SNAPC4 (76) SNDEEDPKDKALPEDPETCLQLNMVYQEVIREKLAEVSQLLAQNQEQQEEILFDLSGTKCPKVKDGRSLPSYMYIGHFLKPYFKDKVTGVGPPANEETRE
hs-SNAPC4 (68) SNDEDDPKDKTLPEDPETCLQLNMVYQEVIQEKLAEANLLAQNREQQEELMRDLAGSKGTKVKDGKSLPPSTYMGHFMKPYFKDKVTGVGPPANEDTRE
dr-SNAPC4 (95) DSSEDSADDLELPQNVETCLQMNLVYQEVLKEKLAELEQLLIENQQQQKEIEVQLSGPGNSIFSVPGVPPQKQFLGYFLKPYFKDKLTGLGPPANEETKE

     (201) 201      210       220       230       240       250       260       270       280       290       300
gg-SNAPC4 (168) KAAQGIKSFEQLLSTKWKSKEKVLLQKSVVSDRLQRLLQPKLLKLSYSNQKLENVKTEMEKQILEKQIKEVEREIEAINQLPESDLLGGRFDEHDWEKIA
mm-SNAPC4 (176) KATQGIKAFEQLLVTKWKHWEKALLRKSVVSDRLQRLLQPKLLKLEYLHEKQSRVSSELERQALEKQIKEAEKEIQDINQLPEEALLGNRLDSHDWEKIS
hs-SNAPC4 (168) KAAQGIKAFEELLVTKWKNWEKALLRKSVVSDRLQRLLQPKLLKLEYLHQKQSKVSSELERQALEKQGREAEKEIQDINQLPEEALLGNRLDSHDWEKIS
dr-SNAPC4 (195) RMKHGSIPVDNLKIKRWEGWQKTLLTNAVARDTMKRMLQPKLSKMEYLSNKLCRAEGEEKEQLKA-QIELIEKQIAEIRTLKDDQLLGDLQDDHDWDKIS

     (301) 301      310       320       330       340       350       360       370       380       390       400
gg-SNAPC4 (268) NVHFDGRRSSEELKKFWQNWEHPNINKNEWTEEETERLKDIAAKRGYLDWQTIAQELGTNRTAFQCLQKYQTYN-KDLKRKEWTRDEDKMLLELVQEMRV
mm-SNAPC4 (276) NINFEGARSAEEIRKFWQSSEHPSISKQEWSTEEVERLKAIAATHGHLEWHLVAEELGTSRSAFQCLQKFQQYN-KTLKRKEWTEEEDHMLTQLVQEMRV
hs-SNAPC4 (268) NINFEGSRSAEEIRKFWQNSEHPSINKQEWSREEEERLQAIAAAHGHLEWQKIAEELGTSRSAFQCLQKFQQHN-KALKRKEWTEEEDRMLTQLVQEMRV
dr-SNAPC4 (294) NIDFEGLRQADDLKRFWQNFLHPSINKSVWKQDEIYKLQAVAEEFKMCHWDKIAEALGTNRTAFMCFQTYQRYISKTFRRTHWTEEEDDLLRELVEKMRI
```

Myb DNA binding domain

```
     (401) 401      410       420       430       440       450       460       470       480       490       500
gg-SNAPC4 (367) GSHIPYKKIAYYMEGRDSAQLIYRWTKSVDPSLKKGPWTPEEDAMLLAAVEKYGERDWYKIRTEVPGRSDAQCSDRYLKALHRDVKKGKWSLKEEEQLID
mm-SNAPC4 (375) GNHIPYRKIVYFMEGRDSMQLIYRWTKSLDPSLKRGFWAPEEDAKLLQAVAKYGAQDWFKIREEVPGRSDAQCRDRYIRRLHFSLKKGRWNAKEEQQLIQ
hs-SNAPC4 (367) GSHIPYRRIVYYMEGRDSMQLIYRWTKSLDPGLKKGYWAPEEDAKLLQAVAKYGEQDWFKIREEVPGRSDAQCRDRYLRRLHFSLKKGRWNLKEEEQLIE
dr-SNAPC4 (394) GNFIPYIQMSHFMVGRDGSQLAYRWTSVLDPSLKKGPWSKEEDQLLRNAVAKYGTREWGRIRTEVPGRTDSACRDRYLDCLRETVKKGTWSYAEMELLKE

     (501) 501      510       520       530       540       550       560       570       580       590       600
gg-SNAPC4 (467) LVQKHGLGHWSKIASELPHRTRSQCLSKWKIMIGSKASRCGKQVLRVTLEDVRRVLRENTCFQR----KR------------------------------
mm-SNAPC4 (475) LIEKYGVGHWARIASELPHRSGSQCLSKWKILARKKQHLQRKRGQRPRHSSQWSSSGSSSSSSEDYGSSSGSDGSSGSENSDVELEASLEKSRALTPQQY
hs-SNAPC4 (467) LIEKYGVGHWAKIASELPHRSGSQCLSKWKIMMGKKQGLRRRR-RRARHSVRWSSTSSSGSSSGSSGGSSSSSSSS-SEEDEPEQAQAGEGDRALLSPQY
dr-SNAPC4 (494) KVAKYGVGKWAKIASEIPNRVDAQCLHKWKLMTRSKKPLKRPLSSITTSYPRNKRQKLLKTVKEEMFFNSSSDDESQINYMNSDESDDLAEDENLEIPQK

     (601) 601      610       620       630       640       650       660       670       680       690       700
gg-SNAPC4 (533) ----------------------------------------------------------------------------------------------------
mm-SNAPC4 (575) RVPDIDLWVPTRLITSQSQREGTGCYPQHPAVSCCT---QDASQNHHKEGSTTVSAAEK--NQLQVPYETHSTVPRGDRFLHFSDTHSASLKDPACKS--
hs-SNAPC4 (565) MVPDMDLWVPARQSTSQPWRGGAGAWLGGPAASLSPPKGSSASQGGSKEASTTAAAPGEETSPVQVPARAHGPVPRSAQASHSADTRPAGAEKQALEGGR
dr-SNAPC4 (594) EYVQTEMKEWIPRNAMVWTITPGSFRTLWVRLPTNEEELRESTKESGLGSDSSENSACPNDEPIXERNTILDRFGDVERTYVGMNTVVLHRRTDDEKA--
```

```
(701)          701       710       720       730       740       750       760       770       780       790       800
gg-SNAPC4  (533) -----------------------------------------TSGVGASAAEKHRGLWSTMGKSRRQERARWRKMNLDRKLLMAVTPWVGDVLLPCTLRAG
mm-SNAPC4  (668) ---------------------HTLMKERPKQPLLPSSRSGSDPGNNTAGPHLRQLWHGTYQNKQRRKRQALHRRLLKHRLLLAVIPWVGDINLACTQAPR
hs-SNAPC4  (665) RLLTVPVETVLRVLRANTAARSCTQKEQLRQPPLPTSSPGVSSGDSVARSHVQWLRHRATQSGQRRWRHALHRRLLNRRLLLAVTPWVGDVVVPCTQASQ
dr-SNAPC4  (692) -----------------------------MFKVC------MSDVKQFIQMKATEFAVKKKKKIKNKKRTLRDVFSLNTDLQKAVIPWIGNVIISTPANEA

(801)          801       810       820       830       840       850       860       870       880       890       900
gg-SNAPC4  (592) KMAFDQTKAYSIQQKLKSVSLSSTPLFTLFIQLFRIDTNGCMKIIRERRADAARPQQPGS-----HLTPDSMLPLTWVVTQQALLSSAVQAVVGVPQGLQ
mm-SNAPC4  (747) RPATVQTKADSIRMQLECARLASTPVFTLLIQLLQIDTAGCMEVVRERKSQPPALLQPGTRNTQPHLLQASSNAKNNTG-CLPSMTGEQTAKRASHKGRP
hs-SNAPC4  (765) RPAVVQTQADGLREQLQQARLASTPVFTLFTQLFHIDTAGCLEVVRERKALPPRLPQAGARDPPVHLLQASSSAQSTPGHLFPNVPAQEASKSASHKGSR
dr-SNAPC4  (757) ----IFCEGDIVGIKAASIRLQKTSVFTFFIKAFHVDVNGCRTVIEIHKKLDIKMPLAINGNP--KPTPISTSPKTVAVLLQQSKAASEHKKPAEPSQQP

                                      Oct-1 Interacting Domain
(901)          901       910       920       930       940       950       960       970       980       990       1000
gg-SNAPC4  (687) AIVRSQSQASVTSSGSVSGLGAPPVSSGVNMPHPSHAETKTDPQLAAGSPAGKTAGVSHSASVFPVSSADPVCSVSGVSSATSAHSDSSSK----TVDSS
mm-SNAPC4  (846) RLGSCRTEATPFQVPVAAPRGLRPKPKTVSELLREKRLRESHAKKATQALGLN-SQLLVSSPVILQPPLLPVPHGSPVVGPATSSVELSVPVAPVMVSSS
hs-SNAPC4  (865) RLASSRVERTLPQASLLASTGPRPKPKTVSELLQEKRLQEARAREATRGPVVLPSQLLVSSSVILQPPLPHTPHGRPAPGPTVLNVPLSGPGAPAAAKPG
dr-SNAPC4  (851) SLPPSQKPSLPPSVPPSQQPTLPPPSQPSQPPPQPPSLPPSQPPAQQPSLPPPQPPSLPPPLPPPQPPSLPPPQPPSLPTSQQQSLPPSQQHSLPPFQNP

(1001)         1001      1010      1020      1030      1040      1050      1060      1070      1080      1090      1100
gg-SNAPC4  (783) AAHNALPGGAPTLHAQLLP--------------------------------QMQLPASTQGSDSRCVTDVVSLGEKQDSSTTNGSSSNSDVLHKGDVLQP
mm-SNAPC4  (945) PSGSWPVGGISATDKQPPN--------------------------------LQTISLNPPHKGTQVAAPAAFRSLALAPGQVPTGGHLSTLGQTSTTSQ-
hs-SNAPC4  (965) TSGSWQEAGTSAKDKRLST--------------------------------MQALPLAPVFSEAEGTAPAASQAPALGPGQISVSCPESGLGQSQAPAAS
dr-SNAPC4  (951) SLPPSQQPSLPPSKQPPQPLPVRQITTPTLIYPNNLVITNPNMEGEVQHLVFKGLLLPQQPSKAVSHIPLPVMQPKTPAQPIVVSKSPSVQDSNSVKSSK

(1101)         1101      1110      1120      1130      1140      1150      1160      1170      1180      1190      1200
gg-SNAPC4  (851) RASIPHNSVAGNSEGSADQGLKYRPIAS-----------------------------------------------------------------K--P---
mm-SNAPC4(1012) -KQSLPKVLPILRAAPSLTQLSVQPPVS----GQPLATKSSLPVNWVL-------------------TTQKLLSVQVPAVVGLPQSVMTPETIGLQAKQL
hs-SNAPC4(1033) RKQGLPEAPPFLPAAPSPTPLPVQPLSLTHIGGPHVATSVPLPVTWVL-------------------TAQGLLPVPVPAVVSLPRPAGTPGPAGLLATLL
dr-SNAPC4(1051) RICKPTKKAQALMEQSKVKSRKKEPQKQNQGNKNVVFPTVTLQTSPVIKILSPARLVQVTGLSPNFSSNQTINMPDKSLTIKSPQPCSSGNLHQSAPVVV

(1201)         1201      1210      1220      1230      1240      1250      1260      1270      1280      1290      1300
gg-SNAPC4  (881) ------------------------PPEQTESAP-------------------------------------------------------------------
mm-SNAPC4(1088) PSPAKTPAFLE------------QPPASTDTEP-------------------------------------------------------------------
hs-SNAPC4(1114) PPLTETRAAQGPRAPALSSSWQPPANMNREPEPSCRTDTPAPPTHALSQSPAEADGSVAFVPGEAQVAREIPEPRTSSHADPPEAEPPWSGRLPAFGGVI
dr-SNAPC4(1151) HSSTNPTFVHSSVSNVSRDNLNVSSTINISPRVSRDALNPTSFLNSTTFPLPQNLSVQQSVQIVPQIPINVVHKATCTKAAKTSSDSSSDESVVKQHQLS

(1301)         1301      1310      1320      1330      1340      1350      1360      1370      1380      1390      1400
gg-SNAPC4  (890) ------------------------------------------------------------------------------------------PQP------T
mm-SNAPC4(1109) ----------------------------------------------------------------------------------------KGPQGQEIPPTP
hs-SNAPC4(1214) PATEPRGTPGSPSGTQEPRGPLGLEKLPL-----------------------------------------------------RQPGPEKGALDLEKPPLP
dr-SNAPC4(1251) PSTGRSIPPAVFNIQPNPSTPPTLSSGPVIFNPNNKVVAPKLCGLNVSSSQLPTVSTQKTKYRPIRPLGPLPVVAPPSRKVTSMSRIRAQSEGEPLISLR
```

SNAPC2 Interacting domain

```
(1401)         1401     1410      1420      1430      1440      1450      1460      1470      1480      1490     1500
gg-SNAPC4 (894) TSSAEKTLLDYSLISLEDDELVKEWLSGKQGVQVPPLQTRLPYFPPFLCNLKTLSKLLLQKAALEKQAARFLSPDGSQTEG---EVDLNAITKLVHEKLG
mm-SNAPC4(1121) --GPEKAALDLSLLSQESEAAIVTWLKGCQGAFVPPLGSRMPYHPPSLCSLRALSSLLLQKQDLEQKASSLAASQAAGAQPDPKAGALQASLELVQRQFR
hs-SNAPC4(1261) QPGPEKGALDLGLLSQEGEAATQQWLGGQRGVRVPLLGSRLPYQPPALCSLRALSGLLLHKKALEHKATSLVVGGEAERP----AGALQASLGLVRGQLQ
dr-SNAPC4(1351) DLPAAGVNFDSHLIFPEKSSEVDDWMDGKGGIPLPHLDTSLPYLPPSAATIKTMTDLLRAKQPLLLAAKKVLPAQYQDECNE--EVEVEAIRKVVAERFA

(1501)         1501     1510      1520      1530      1540      1550      1560      1570      1580      1590      1600
gg-SNAPC4 (991) DDPAFLLLKARFLAAFTLPAVLATLPPPKVATTLSASKREYGESDEEEWQSEEEASEDESCGDELDAPSDETGGDEPGDRDADFPSKGMETEDSAAQSIV
mm-SNAPC4(1219) DNPAYLLLKTRFLAIFSLPAFLATLPPNSIPTTLSPDVAVVSES-DSEDLGDLELKDRARQLDCMACRVQASPAAPDPVQRAPSPG-----EVSAPSPLD
hs-SNAPC4(1357) DNPAYLLLRARFLAAFTLPALLATLAPQGVRTTLSVPSRVGSESEDEDLLSELELADRDGQP--------GCTTATCPIQGAPDSG-----KCSASSCLD
dr-SNAPC4(1449) SNPAYLLCKARFLSCFTLPALLATINPCEERQLLSEDDEEDDHLATINPSEEHQSSTEDDEEDLQTNERSQPPTARTELNMNENEA--------------

(1601)         1601     1610              1629
gg-SNAPC4(1091) GTCTDGTASVPQIRRSVRIRKRFTKRRRV
mm-SNAPC4(1313) ASDGLDDLNVLRTRRARHSRR--------
hs-SNAPC4(1444) TSNDPDDLDVLRTRHARHTRKRRRLV---
dr-SNAPC4(1535) -SAKQFSGIGPKRQRNQRIKRLIK-----
```
