# Supplementary material for: Fast Homozygosity Mapping and Identification of a Zebrafish ENU-Induced Mutation by Whole-Genome Sequencing
Source: PLoS One. 2012 Apr 4;7(4):e34671. doi: 10.1371/journal.pone.0034671 (PMC3319596; doi:10.1371/journal.pone.0034671)
Supplement: Table S1 — List of the SNPs within the A, B, C and D homozygosity regions on chromosome 5 that create an aminoacid change and are specific to the m1045 genome. The position of the SNP on the chromosome 5 is indicated, the codon affected and the type of substitutions: the column “aa reference" corresponds to the aa found in the Tü genome while the column “aa m1045" correspond to the aa found in the m1045 genome. 30 SNPs create missense variations compared to the reference while only one SNP (underlined in yellow) creates a STOP codon in the transcripts ENSDART00000097473 and ENSDART00000141424, coding for the Snapc4 protein. (PDF) [file pone.0034671.s006.pdf]

Accession Number	Chr	Position	N° codon	Codon reference	Codon m1045	aa reference	aa m1045
ENSDART00000132406	chr5	14675032	148	GGG	AGG	G	R
ENSDART00000140769	chr5	34474899	22	TCG	TTG	S	L
ENSDART00000145805	chr5	39768799	133	CTT	TTT	L	F
ENSDART00000137658	chr5	40346394	113	ATA	ACA	I	T
ENSDART00000004286	chr5	40689935	96	AAC	GAC	N	D
ENSDART00000051256	chr5	41143173	8	TTT	TTG	F	L
ENSDART00000083294	chr5	52558074	39	TCT	TTT	S	F
ENSDART00000128402	chr5	52575792	367	TCA	TTA	S	L
ENSDART00000083294	chr5	52583676	1126	GTA	TTA	V	L
ENSDART00000083317	chr5	52592819	382	CAA	CGA	Q	R
ENSDART00000097460	chr5	52671348	428	CCG	TCG	P	S
ENSDART00000149553	chr5	52671348	428	CCG	TCG	P	S
ENSDART00000097460	chr5	52671546	389	GTT	ATT	V	I
ENSDART00000149553	chr5	52671546	389	GTT	ATT	V	I
ENSDART00000074638	chr5	52689025	119	TTT	CTT	F	L
ENSDART00000097466	chr5	52776118	200	GTT	TTT	V	F
ENSDART00000110377	chr5	52788734	160	CGC	CAC	R	H
ENSDART00000110377	chr5	52789075	274	TTG	ATG	L	M
ENSDART00000110377	chr5	52789529	425	AGT	AAT	S	N
ENSDART00000110377	chr5	52789610	452	GCA	GGA	A	G
ENSDART00000110377	chr5	52789933	560	GAA	AAA	E	K
ENSDART00000110377	chr5	52790383	710	GAA	AAA	E	K
ENSDART00000092002	chr5	52800436	179	TTT	TCT	F	S
ENSDART00000092002	chr5	52802749	224	CAA	CGA	Q	R
ENSDART00000092002	chr5	52807088	366	AAA	AGA	K	R
ENSDART00000092002	chr5	52811906	592	CTA	ATA	L	I
ENSDART00000130660	chr5	52814562	1463	CGG	CAG	R	Q
ENSDART00000042420	chr5	52814562	1297	CGG	CAG	R	Q
ENSDART00000130660	chr5	52823229	1076	AAC	GAC	N	D
ENSDART00000042420	chr5	52823229	925	AAC	GAC	N	D
ENSDART00000130660	chr5	52829530	743	AGT	ATT	S	I
ENSDART00000130660	chr5	52829542	739	ATG	ACG	M	T
ENSDART00000130660	chr5	52830849	493	AAG	AGG	K	R
ENSDART00000042420	chr5	52830849	404	AGT	GGT	S	G
ENSDART00000130660	chr5	52835790	99	TAT	AAT	Y	N
ENSDART00000042420	chr5	52835790	99	TAT	AAT	Y	N
ENSDART00000111120	chr5	52835790	17	TAT	AAT	Y	N
ENSDART00000130660	chr5	52836035	49	AAT	AGT	N	S
ENSDART00000042420	chr5	52836035	49	AAT	AGT	N	S
ENSDART00000141424	chr5	52876809	125	TGG	TGA	W	STOP
ENSDART00000097473	chr5	52876809	117	TGG	TGA	W	STOP
